# Strain Hardening of Polypropylene Microfiber Reinforced Composite Based on Alkali-Activated Slag Matrix

**DOI:** 10.3390/ma15041607

**Published:** 2022-02-21

**Authors:** Olga M. Smirnova, Ignacio Menendez Pidal, Aleksandr V. Alekseev, Dmitry N. Petrov, Mikhail G. Popov

**Affiliations:** 1 Department of Constructing Mining Enterprises and Underground Structures, Saint-Petersburg Mining University, 21-st Line V.O., 2, 199106 Saint-Petersburg, Russia; a1exeev@yandex.ru (A.V.A.); petrovgs@mail.ru (D.N.P.); popov_mg@pers.spmi.ru (M.G.P.); 2 Laboratorio de Geología, Departamento de Ingeniería y Morfología del Terreno, Universidad Politécnica de Madrid, 28040 Madrid, Spain; ignacio.menendezpidal@upm.es

**Keywords:** sustainability, rheologically active inorganic filler, stress–strain deformation curve, acoustic emission technique, acoustic event, monitoring of structures

## Abstract

A comparative study of the fracture features, strength and deformation properties of pseudo strain-hardening composites based on alkali-activated slag and Portland cement matrices with polypropylene microfiber was carried out. Correlations between their compositions and characteristics of stress–strain diagrams under tension in bending with an additional determination of acoustic emission parameters were determined. An average strength alkali-activated slag matrix with compressive strength of 40 MPa and a high-strength Portland cement matrix with compressive strength of 70 MPa were used. The matrix compositions were selected for high filling the composites with polypropylene microfiber in the amount of 5%-vol. and 3.5%-vol. ensuring the workability at the low water-to-binder ratios of 0.22 and 0.3 for Portland cement and alkali-activated slag matrices, respectively. Deformation diagrams were obtained for all studied compositions. Peaks in the number of acoustic signals in alkali-activated slag composites were observed only in the strain-softening zone. Graphs of dependence of the rate of acoustic events occurrence in samples from the start of the test experimentally prove that this method of non-destructive testing can be used to monitor structures based on strain-hardening composites.

## 1. Introduction

Recently attention is paid to the development of new compositions of cement matrices and the use of modified multicomponent fibers in strain-hardening cement composites subjected to static and dynamic loads [1,2,3,4] since construction practice shows a high demand for mineral composites with special deformation characteristics [5,6,7,8].

The important advantages of such composites are the significant increase (up to 40% or more) of the value of tensile stresses corresponding to the sample destruction compared with the value of tensile stresses corresponding to the first microcrack and sufficiently large relative deformations of cement composite under uniaxial tension (up to values of several percent) due to a significant increase of the volume of microcracking before destruction [9,10,11].

A high relative deformation of up to 5% is the result of the sequential formation of multiple, densely spaced microcracks under increasing uniaxial tensile loading [12]. This behavior of the material under tension is achieved by fulfilling a number of micromechanical conditions for the formation of microcracks, their propagation and overlap with fibers [13,14,15]. This cement composite can be used not only as the main material in structures but also as thin reinforcing layers for repairing and strengthening the existing structures taking into consideration its high deformation ability [16,17,18,19]. Another advantage of these composites is the use of secondary mineral resources, such as microsilica, slag, fly ash, ground rocks, or stone dust [20,21,22,23,24]. The application scope of a strain-hardening cement composite (SHCC) depends on its strength and deformation characteristics [25,26,27,28,29,30]. The values of these characteristics are determined by properties of the cement matrix, microfiber, as well as by properties of the contact zone between matrix and microfiber [29,30]. Microcracking, namely the number and width of cracks determines the pseudoplasticity of composites and affects their durability that depends on the composite permeability [31,32].

In this paper, the high-strength cement matrix with a compressive strength of 70 MPa based on Portland cement and an average strength matrix with compressive strength of 40 MPa based on ground granulated blast furnace slag with alkaline activation were used. Polypropylene microfiber was used as reinforcement. Polypropylene microfiber (PP) is a widespread and affordable material since there is an overproduction of polypropylene in the world [33]. Studies on the use of PP fibers in the amount of 0.1–0.5% in cement composites are continuing, for example, reduction of shrinkage of concrete based on seawater and sea sand is shown in [34]. The strain-hardening behavior of composites based on inorganic binders with PP microfiber has not been sufficiently studied.

Cement composites with 2%-vol. of PP microfiber have shown a tensile strain capacity in the range of 3–6%, with a first cracking strength of 1.6–2.1 MPa, an ultimate tensile strength of 2.2–2.8 MPa and compressive strength of 15–45 MPa. These new composites are significantly more ductile with higher tensile strain capacity at least 300 times more than the tensile strain of reference composite without PP microfibers [35]. The addition of low-modulus hydrophobic PP microfiber has improved the ductility and the strain-hardening behavior of the cement composites containing polyvinyl-alcohol (PVA) microfiber [36].

The use of industrial by-products gives economic and environmental benefits [37,38,39]. The mechanical properties of alkali-activated ladle slag mortars reinforced by multifilament-PP fiber (PP-MF) or split-film-PP fiber (PP-SF) in the amount of 2%-vol. reveals that the effect of PP microfibers is evident with an increase of up to 300%, 80%, 7.6 times and 150 times of flexural strength, tensile strength, fracture toughness and fracture energy, respectively. Furthermore, the PP-SF microfiber offers a better mechanical response than the PP-MF microfiber in post peak load carrying capacity of the reinforcement at uniaxial tension. Pseudo strain-hardening behavior was observed along with multiple microcracks under the uniaxial tensile test [40].

The mechanical properties of composite based on ladle slag and gypsum (LSG) were significantly improved by using the PP microfiber as reinforcement. The LSG composites with the 2%-vol. of PP microfiber had pseudo strain-hardening behavior and high ductility. Multiple microcracks along with pseudo strain-hardening behavior were observed by the DIC technique under uniaxial loading. The mechanical properties of 2%-PP-LSG mortars have increased up to 130%, 40%, 5.30 times and 124.8 times for flexural strength, compressive strength, fracture toughness and fracture energy, respectively, after 28 days of curing in a water bath [41].

The partial replacement of PVA microfiber with PP microfiber in cement composites with 1.2%, 1.5% and 2% of PVA microfiber was studied in [42]. The PP microfibers with different cross-sectional shapes were used in the amount of 25 and 40 vol.% of the PVA microfiber. It was found that PP microfiber with non-round cross-sectional shapes (i.e., triangular and trilobal) considerably improved the deformability of composites under the bending load but reasonably decreased the flexural strength of composites. The main effect of low modulus PP fibers was the improvement of composite deformability which increased the energy absorption capacity up to 75%. The results indicated that cementitious composites with moderate strength, high deformability and lower cost can be obtained using the replacement of PVA fiber with non-round low modulus PP fiber [42].

From the analysis of the published results, it can be concluded that the use of PP microfiber contributes to the enhancement of ductility, but the results vary significantly. The difference in the mechanical properties of PP microfiber may be one of the reasons. The microfiber manufacturers can add fillers and other modifying additives that lead to a change of properties of PP microfiber. PP microfibers properties of the above-cited papers are presented in Table 1.

A method is needed to assess the strength and deformation properties of the material in the structure under operating conditions [43,44,45]. The analysis of deformation curves and acoustic emission parameters can be used to evaluate the destruction of composites. Acoustic emission (AE) testing is a technique to detect the formation and growth of cracks both on the surface and inside the material. Crack formation and propagation in brittle materials are combined with a local rapid release of stored strain energy. The resulting elastic waves propagate through the material and can be detected by piezoelectric sensors on the surface. The characteristics of the elastic waves and hence the electric signals from the sensors depend on the crack type and the acoustic properties of the material [46]. Analysis of the signal parameters (e.g., amplitude and average frequency) makes it possible to classify the cracks, e.g., as described in papers [47,48,49]. In combination with the stress–strain curves one can draw conclusions about the destruction mechanism of SHCCs [46,50].

The aim of the paper is to study the strain-hardening behavior under tension in bending of composites based on inorganic binders with polypropylene microfiber with the additional determination of acoustic emission parameters.

## 2. Materials and Methods

### 2.1. Materials and Mixture Composition

Ordinary Portland cement (CEM I 42.5) was used for the study. The microfillers were obtained by grinding quartz sand and marking it as A1.5 (D_50_ = 1.5 µm) and A4 (D_50_ = 4 µm). The ground quartz sands with different fineness were marked as A1.5; A4; A15. The properties of polypropylene (PP) microfiber are presented in Table 2. Mix proportions are presented in Table 3 and Table 4.

The compositions based on Portland cement differed in the microfiber amount. The compositions based on alkali-activated slag binder differed in the water-to-binder ratio.

### 2.2. Manufacturing Procedure

**Figure 1:**
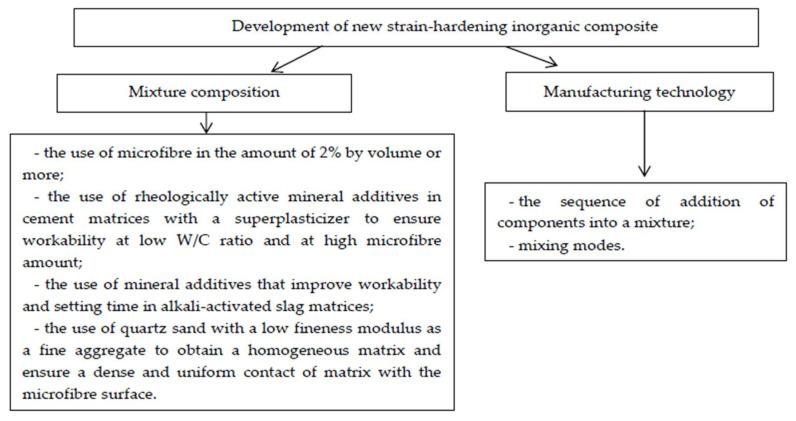
Scheme for the development of new compositions of SHCCs.

The workability of the fresh mixture decreases with an increase in the microfiber quantity. Summarizing the experience of other scientists and own experience it is advisable to develop new compositions of strain-hardening of inorganic composite according to the scheme in Figure 1. Development of new strain-hardening inorganic composite can be in mixture composition and in manufacturing technology. In study of mixture compositions the following results were obtained: the use of microfiber in the amount of 2% by volume or more [51,52,53,54,55]; the use of rheologically active mineral additives in cement matrices with a superplasticizer to ensure workability at low W/C ratio and at high microfiber amount [56,57]; the use of mineral additives that improve workability and setting time in alkali-activated slag matrices [58,59,60,61]; the use of quartz sand with a low fineness modulus as a fine aggregate to obtain a homogeneous matrix and ensure a dense and uniform contact of matrix with the microfiber surface [62,63,64,65,66,67,68]. Manufacturing technology takes into account: the sequence of addition of components into a mixture [69,70,71,72,73]; mixing modes [62,74,75,76,77].

It is shown in papers [1,2,3,62] that it is necessary to increase the matrix density to achieve strain-hardening in composites with hydrophobic microfiber. An increase in density can be achieved by reducing the water-to-cement ratio using a superplasticizer and rheologically active mineral additives. High filling with microfiber can be achieved by justifying the choice of the type of concrete mixer [68,69,70,71], the sequence of addition of components [72,73,74], and the speed and duration of mixing [62,75,76,77].

**Figure 2:**
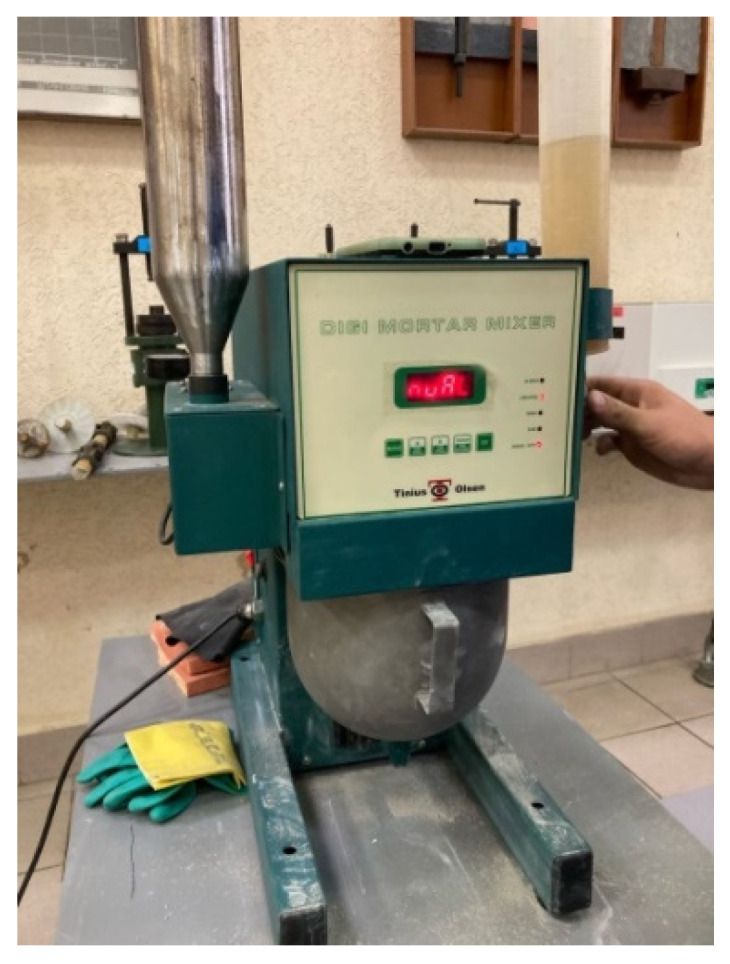
Tinius Olsen laboratory automatic mixer.

A laboratory automatic mixer for mortars from Tinius Olsen was used to prepare the mixtures (Figure 2). It has three mixing modes with different speeds.

At first, the fresh mixture was made without fiber. In both cases at preparing the cement matrix or the slag-alkali matrix, the fresh mixtures were obtained with high fluidity despite the low values of the water-to-cement ratio. This was achieved by taking into account the recommendations from Figure 1. Then the PP microfiber was added and the mixer was started at the second speed for 180 s. Composition of matrices with maximum microfiber quantity (5.5 and 3.5%) was selected taking into account the mixture workability. The spread diameter of mixtures on the shaking table was 160 mm after 15 shakes.

**Figure 3:**
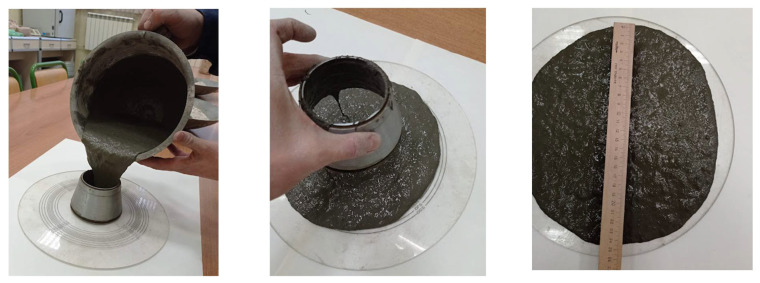
Workability of cement mixture with 2.5% vol. of PP microfiber.

A cement mixture with 2.5% vol. of PP microfiber is shown in Figure 3. This cement mixture was very fluid and made it possible to increase the microfiber amount up to 5.5% by volume.

### 2.3. Experimental Devices and Procedures

#### 2.3.1. Mechanical Properties

**Figure 4:**
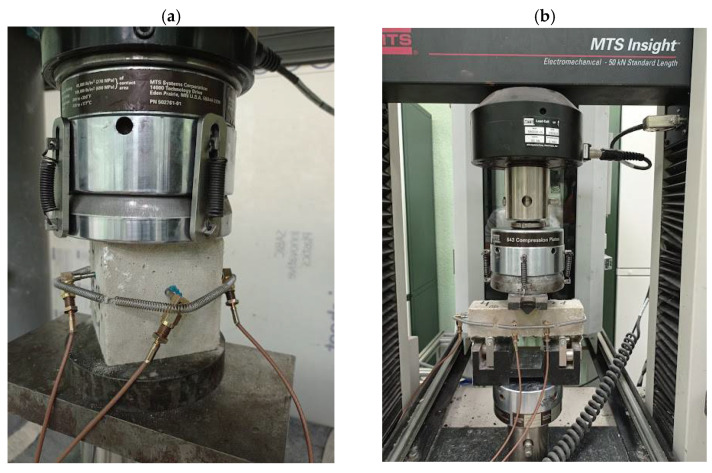
MTS servo-hydraulic system: (**a**) compression test; (**b**) three-point bending test.

The study to determine the mechanical properties was carried out using the MTS 816 servo-hydraulic system that tests samples at compression and three-point bending (Figure 4). Three specimens were tested for each bath at the age of 28 days. The cube samples had a side of 7.07 cm; the beam samples had dimensions of 4 × 4 × 16 cm.

The calculation of the specific fracture work was performed as the ratio of the area under the deformation curve to sample volume. Strain-hardening after the formation of the first microcrack was defined as the difference between the strength corresponding to the sample destruction and the strength corresponding to the first microcrack.

#### 2.3.2. Acoustic Emission (AE)

A set of equipment was used to study the acoustic emission (AE) of samples consisting of the following main parts: MTS 816 servo-hydraulic system; ErgoTech acoustic emission control system; data collection and processing system, and specialized ASC software.

**Figure 5:**
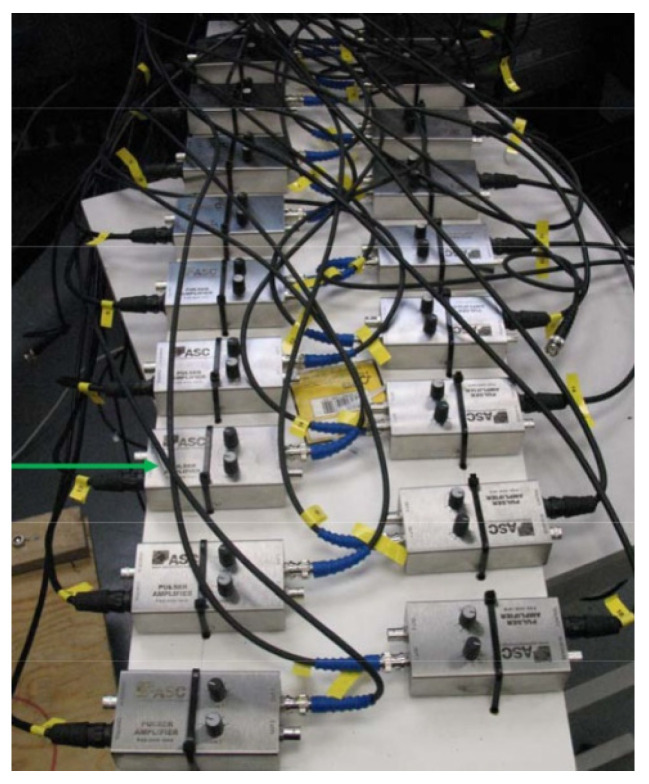
ErgoTech acoustic signal preamplifier unit.

The ErgoTech acoustic rock emission system makes it possible to study the processes of micro- and macro-cracking in rocks under complex loading conditions using acoustic emission sensors. The ErgoTech acoustic emission control system includes a measuring unit with sensors; an acoustic signal preamplifier (Figure 5) for signal amplification and transmission to the information acquisition system; a unit for generating, collecting and processing acoustic signals.

The data collection and processing system, as well as the specialized software of ASC company, are designed for automated control of the process of data registration from sensors in the mode of constant and trigger control, waveform configuration, digital signal processing and filtering, localization and visualization of the received data in graphical and digital form. The data collection and processing system is made in the form of a server.

The type of sensor was a piezoelectric inducer with a 1.3 MHz resonant frequency (Ergotech manufacturer). Preamplifier type was ASC Pulser Amplifier (PAD-006). Lower and upper thresholds were 100 kHz and 2 MHz, accordingly. Calibration of sensors was performed by the Ergotech manufacturer. Calibration by the Nelson method (fracture of the lead) was performed before the tests.

## 3. Results

### 3.1. Compressive Strength and Tensile Strength in Bending

The mechanical properties of four types of composites are summarized in Table 5 including the compressive strength and the tensile strength in bending at the age of 28 days.

The results of Table 5 show that the increase of PP microfiber amount from 4.4% to 5.5% by volume in cement composites led to a decrease in bending strength. This contradicts the well-known pattern that the bending strength increases with increasing the microfiber quantity. However, this pattern has been stated for microfiber quantity in the range from 0 to 2.5% by volume [15,64]. If polypropylene microfiber is used in larger quantities (5.5% by volume) the interface area between microfiber and matrix increases and, possibly, the strength of the contact zone between microfiber and cement matrix begins to prevail.

**Figure 6:**
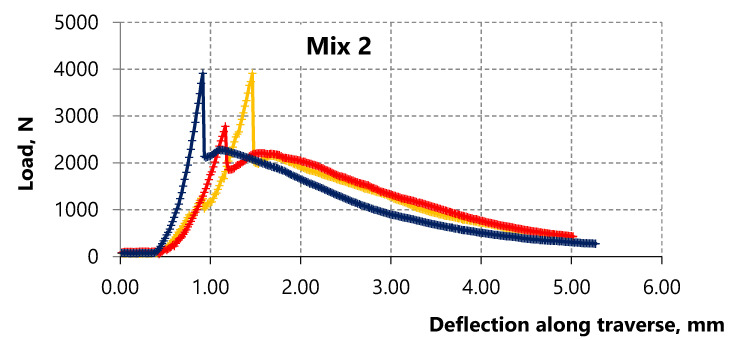
Deformation curves of three samples of Portland cement composite with 4.4% of PP microfiber.

**Figure 7:**
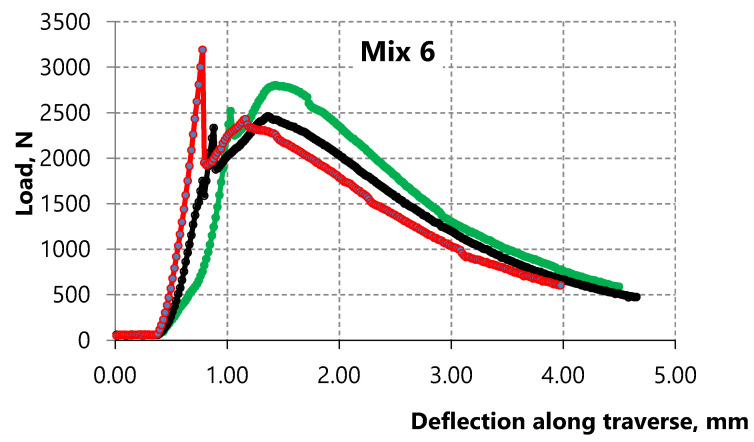
Deformation curves of three samples of Portland cement composite with 5.5% of PP microfiber.

The surface of PP microfiber is hydrophobic so there is no chemical interaction between the surface of this fiber and the cement matrix. In this case, the strength of the contact zone between the PP microfiber and cement matrix is due to friction forces. Hence, the strength of the contact zone between the microfiber and cement matrix, with the increase of PP microfiber amount, affects the bending strength corresponding to the first microcrack in the matrix, namely, reducing it. Accordingly, the bending strength of the 6 Mix with 5.5% fiber is lower than the bending strength of the 2 Mix with 4.4% fiber as shown in Figure 6 and Figure 7. The strain-hardening zone appears on the curves of both mixes. However, this zone in samples of the 6 Mix is of interest because the bending strength corresponding to the sample destruction exceeds the bending strength corresponding to the first microcrack.

**Figure 8:**
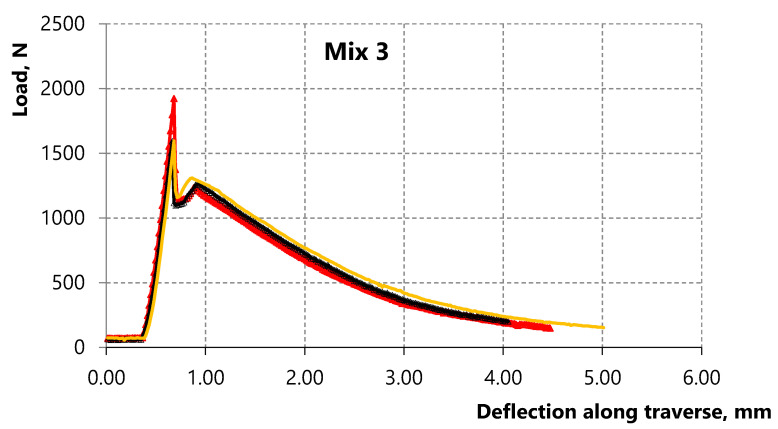
Deformation curves of three samples of alkali-activated slag composite with 3.5% vol. of PP microfiber and W/B = 0.3.

**Figure 9:**
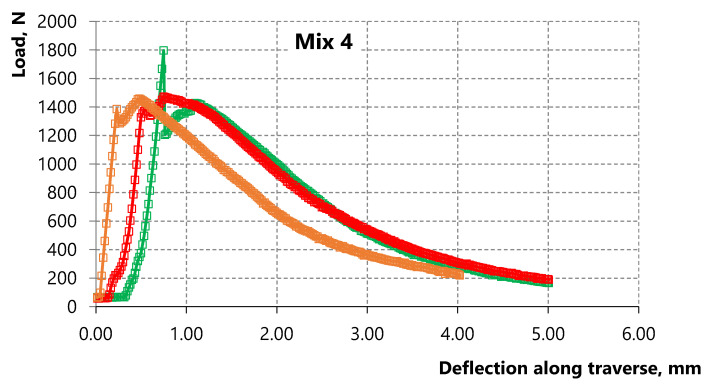
Deformation curves of three samples of alkali-activated slag composite with 3.5% vol. of PP microfiber and W/B = 0.22.

The results show a contradictory pattern in the case of composites with alkali-activated slag matrix (compositions 3 and 4). The bending strength decreases with the decrease of the water-to-binder ratio at a constant microfiber amount according to Table 5 and Figure 8 and Figure 9. However, the strain-hardening zone appears in composition 4 with W/B = 0.22, which is more pronounced compared to the zone on the deformation curve of composition 3 with W/B = 0.3, namely, the bending strength corresponding to sample destruction exceeds the bending strength corresponding to the first microcrack. Thus, strain-hardening behavior in which the strength value corresponding to sample destruction exceeds the strength value corresponding to the first crack appears in the studied alkali-activated slag matrix of medium strength with the decrease of the water-to-binder ratio up to 0.22.

Thus, the effect of the PP microfiber amount and the water-to-binder ratio is studied in this paper. Analysis of sample destruction showed that the characteristics of deformation curves significantly depend on the compositions. In general, the low-strain hardening composites and the high-strain hardening composites can be distinguished depending on the nature of deformation [78].

When operating construction structures made on the basis of strain-hardening composites reliable methods of monitoring changes in mechanical and deformation properties are needed, for example, the acoustic emission method.

### 3.2. AE Measurements

Flow characteristics, such as the number of signals and activity are used to describe the patterns of AE behavior during sample loading [79,80]. The signal number of AE is registered for a certain time interval that counts from the beginning of observation. The activity is a derivative of the number of signals in time. Activity shows an increase in the number of AE signals registered per unit of time.

**Figure 10:**
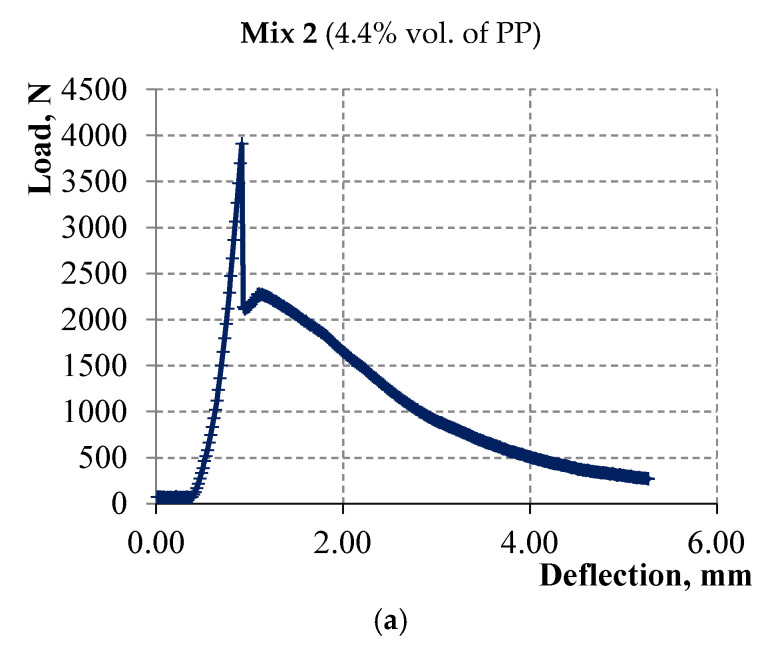

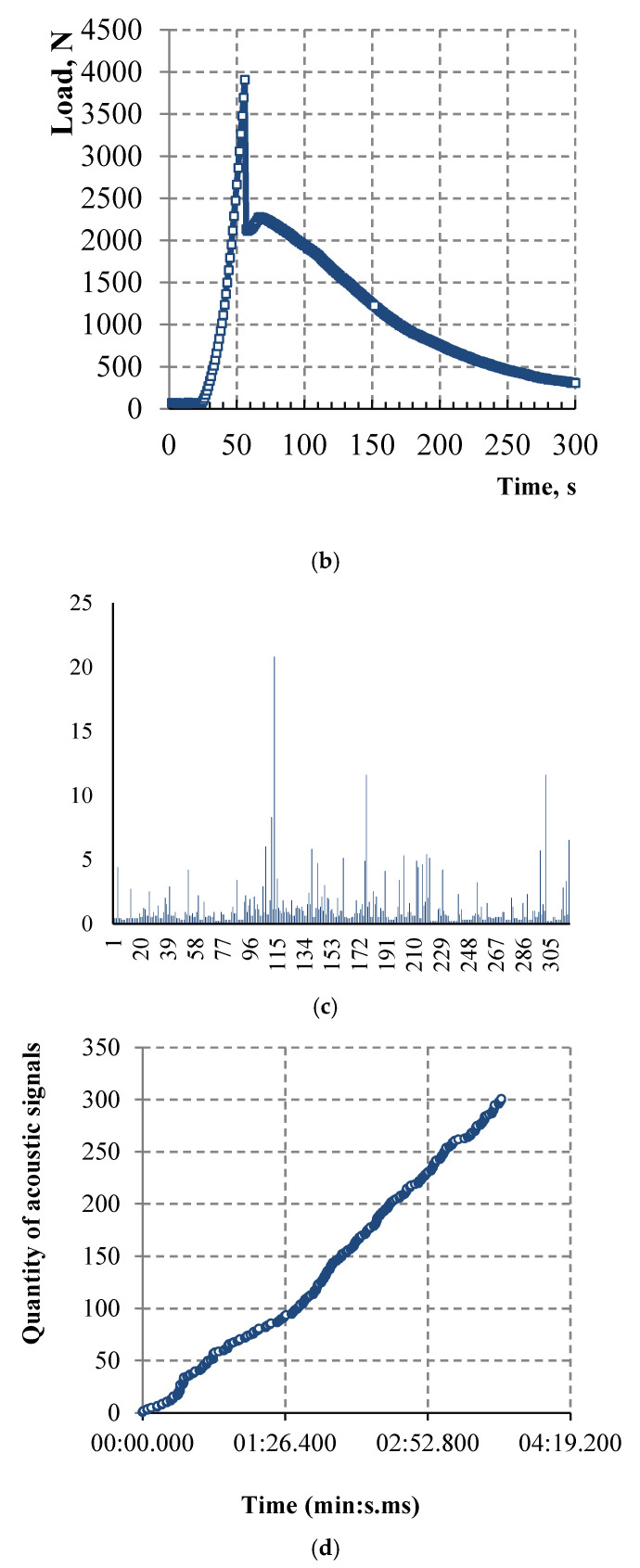
(**a**) Deformation curve; (**b**) Loading speed; (**c**) The number of acoustic signals per second in the range from 0 to 325 s; (**d**) The total number of acoustic signals from the start of the test.

**Figure 11:**
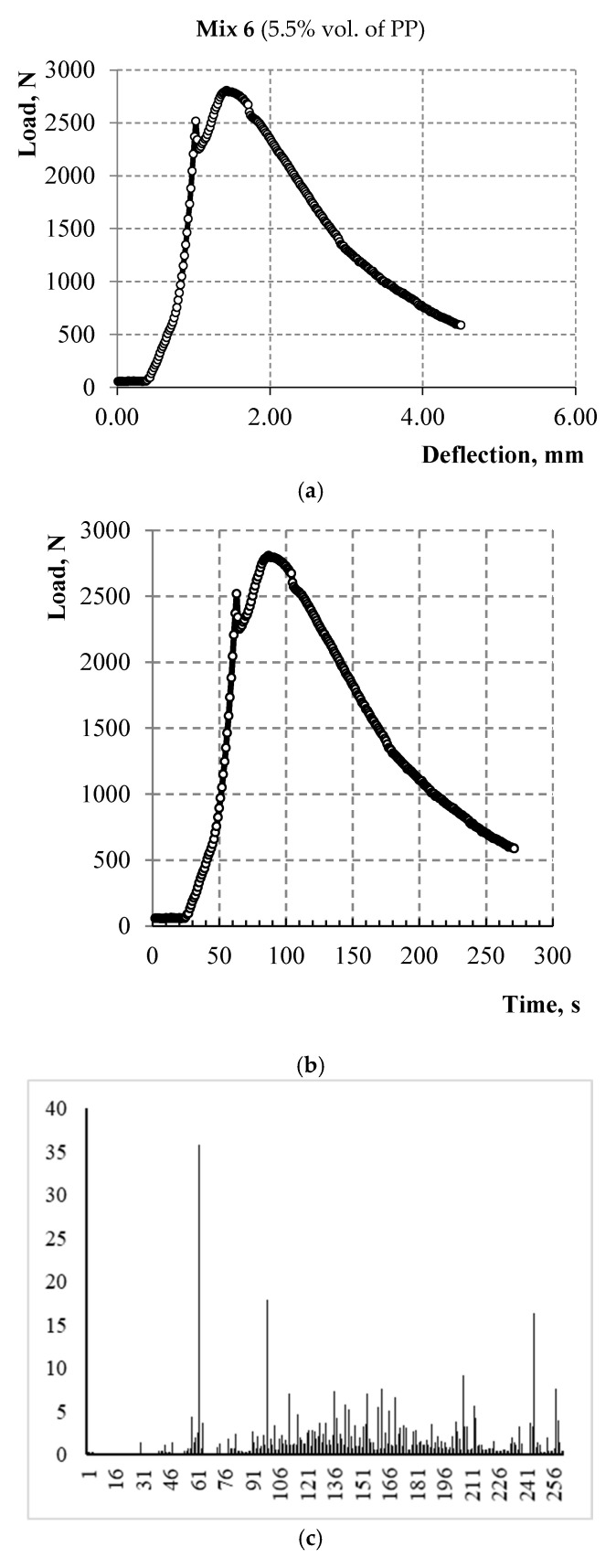

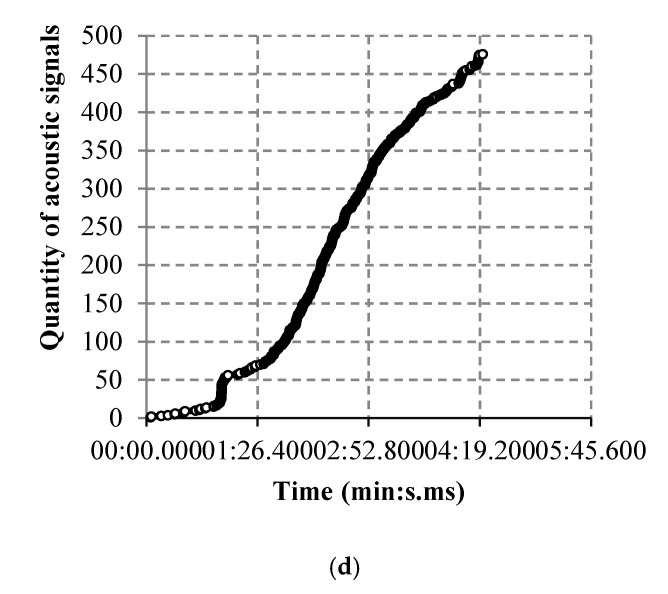
(**a**) Deformation curve; (**b**) Loading speed; (**c**) The number of acoustic signals per second in the range from 0 to 260 s; (**d**) The total number of acoustic signals from the start of the test.

The linkage of the flow characteristics of acoustic emission with the loading process has been studied and is shown in Figure 10 and Figure 11 for a sample of cement-based composite with 4.4% vol. of PP microfiber and for a sample of slag-alkali-based composite with 5.5% vol. of PP microfiber, respectively. The diagrams “load—strain on the traverse” are shown in Figure 10a and Figure 11a. The load speed is shown in Figure 10b and Figure 11b up to the deformation equal to 5 mm or up to the moment of sample destruction. The number of recorded acoustic signals per second in the time interval at which the load speed is indicated is shown in Figure 10c and Figure 11c. The total number of acoustic signals from the start of the test is shown in Figure 10d and Figure 11d.

Each of the presented materials has deformation features. The strain-hardening zone is observed on the deformation diagram of cement composite with 5.5% vol. of PP microfiber. A sharp increase in the number of acoustic signals is observed at the beginning and end of the strain-hardening zone in Figure 11b,c. A strain-hardening zone is almost absent in cement composite with 4.4% vol. of PP microfiber. Several splashes of acoustic signals are observed in this sample only in the strain-softening zone.

Stable relationships between mechanical and AE parameters are observed in the above dependencies despite significant differences in the mechanical characteristics of the studied cement-based composites.

**Figure 12:**
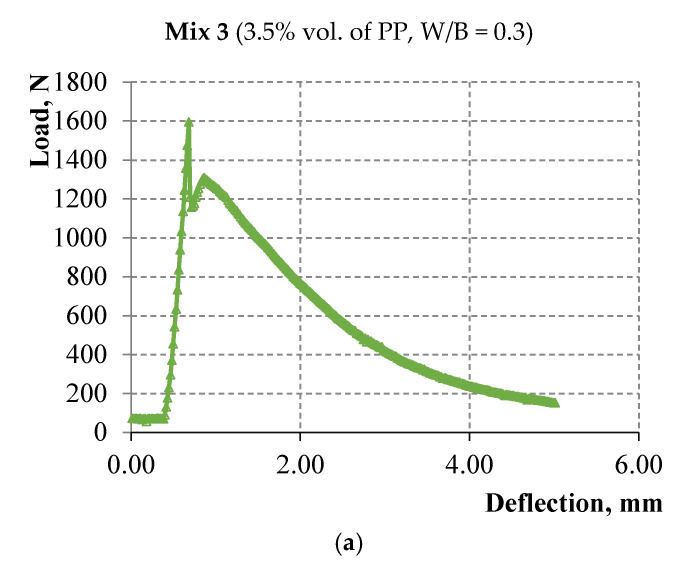

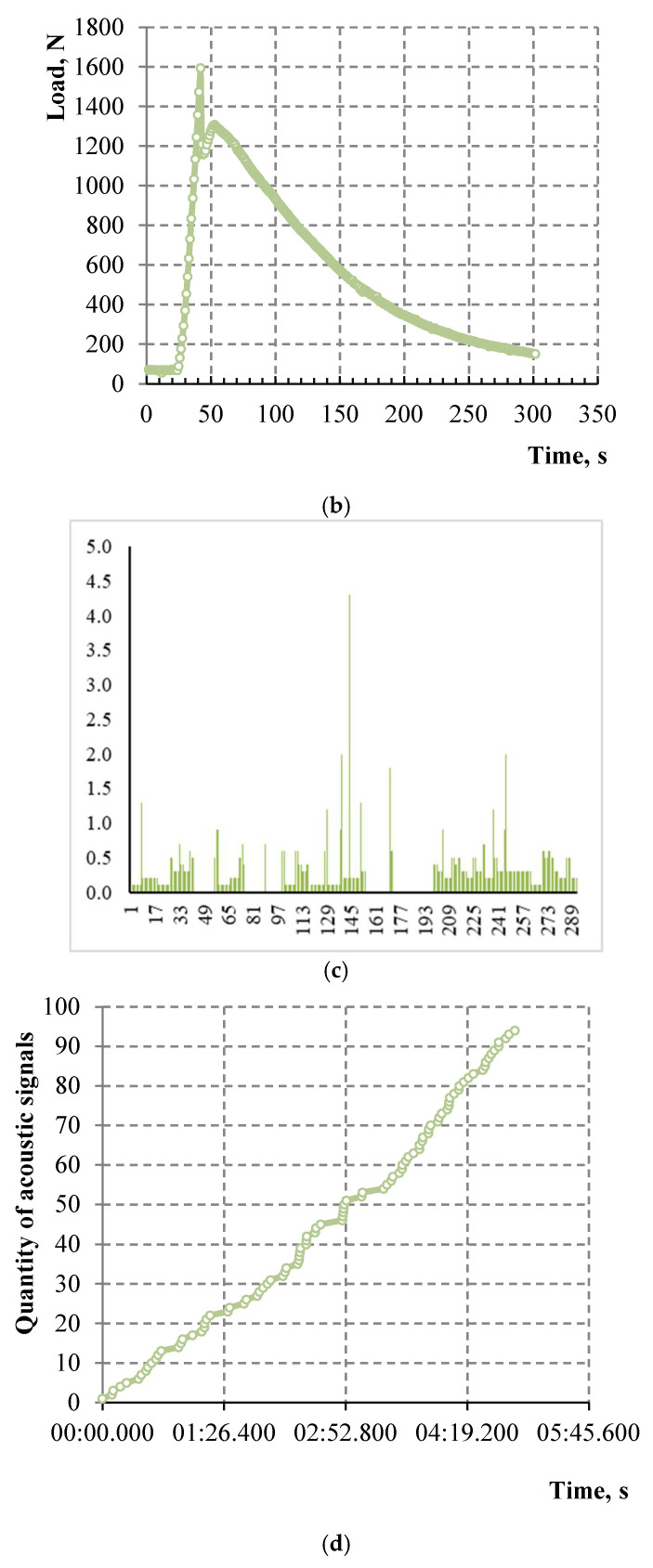
(**a**) Deformation curve; (**b**) Loading speed; (**c**) The number of acoustic signals per second in the range from 0 to 300 s; (**d**) The total number of acoustic signals from the start of the test.

**Figure 13:**
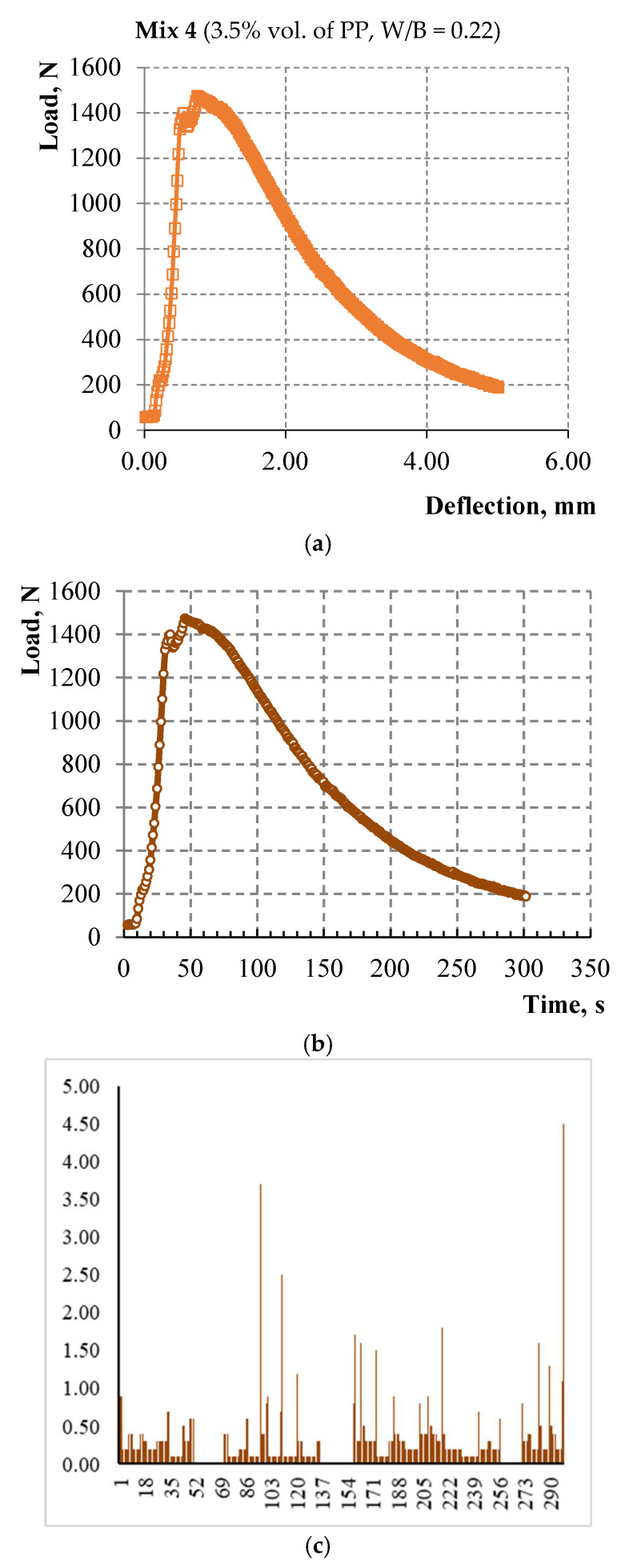

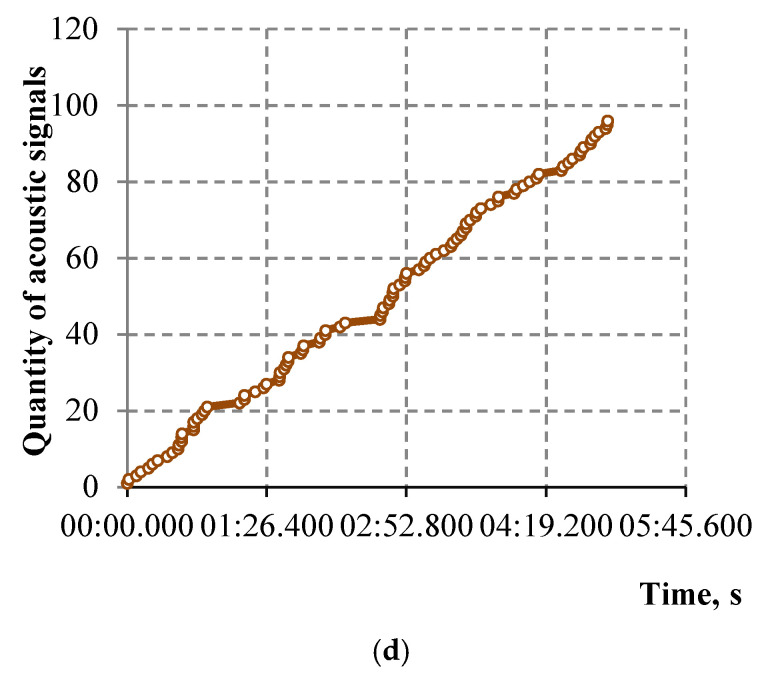
(**a**) Deformation curve; (**b**) Loading speed; (**c**) The number of acoustic signals per second in the range from 0 to 300 s; (**d**) The total number of acoustic signals from the start of the test.

The relationship of flow characteristics of the acoustic emission with the loading curve has been studied and is shown in Figure 12 and Figure 13 for alkali-activated slag based composites with 3.5% vol. of PP microfiber and with different W/C ratios. The diagrams “load—strain on the traverse” are shown in Figure 12a and Figure 13a. The load speed is shown in Figure 12b and Figure 13b up to the deformation equal to 5 mm or up to the moment of sample destruction. The number of recorded acoustic signals per second in the time interval at which the load speed is indicated is shown in Figure 12c and Figure 13c. The total number of acoustic signals from the start of the test is shown in Figure 12d and Figure 13d.

A smaller number of acoustic signals by 3–4 times is typical for samples on alkali-activated slag binder. Peaks in the number of acoustic signals in these samples were observed only in the strain-softening zone.

Graphs of dependence of the occurrence rate of acoustic signals (the first derivative) in the sample from the start of the test, obtained in the work, experimentally prove that this method of non-destructive testing can be used to monitor structures based on strain-hardening composites operating in difficult conditions. These graphs make it possible to use the AE method to estimate the residual resource of the material during deformation.

In further studies linking the AE parameters with microscopic fracture parameters, it is necessary to formulate a number of diagnostic AE indicators for monitoring and evaluating the resource of material.

## 4. Discussion

The characteristics of the studied materials obtained from deformation curves are presented in Table 6.

**Figure 14:**
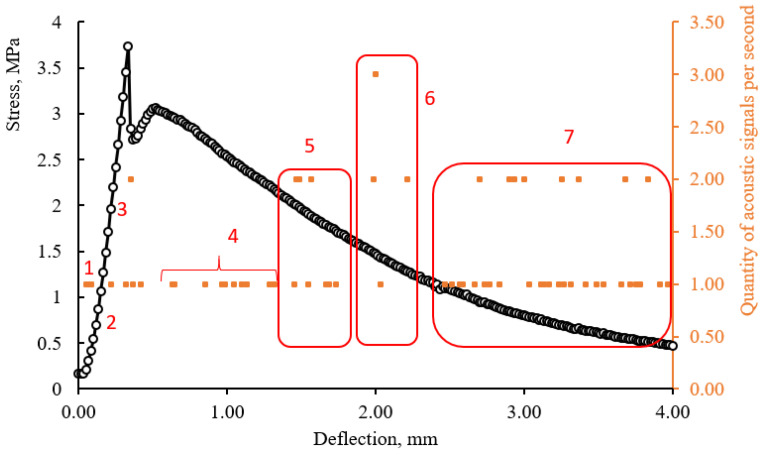
Combined graphs of the dependence of stresses and the number of acoustic events on deflections (Mix 3).

**Figure 15:**
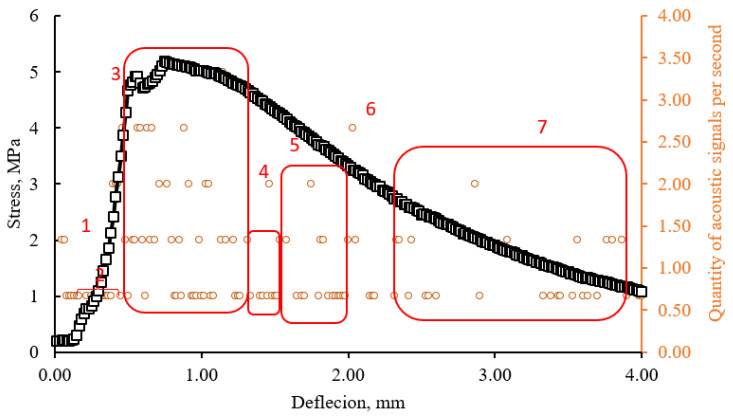
Combined graphs of the dependence of stresses and the number of acoustic events on deflections (Mix 6).

According to the study of beam samples in three-point bending with controlled deformations, it was found that the frequency of occurrence of acoustic events is associated with the deformation curve. The recorded acoustic signals can be combined into seven groups depending on the stage of sample deformation as shown in Figure 14 and Figure 15. These seven groups are outlined with red lines that limit the areas where the values of the number of acoustic signals per second are grouped. At the initial stage of loading, as the traverse displacement increases, the accumulation of stresses occurs in the stretched and compressed zone of a beam-sample. The periodic appearance of signals is typical for this zone (1). Zones 1 and 2 have a very conditional separation and are determined by the nature of deformation of a beam-sample at the initial stage during compression and stretching deformations in a matrix. The duration of signal registration is determined by the fracture work and the elasticity modulus of the material. A drastic increase of acoustic events per second is observed in zone (3), while the size of this zone is associated with the potential for strain-hardening. The formation of microcracks begins and for this reason the dependence of “stress–strain” becomes nonlinear. The frequency of signals per unit of time in this zone indicates the trajectory of destruction: with an instantaneous burst of a series of signals the destruction occurs according to a fragile scenario, with a periodic, prolonged appearance of signals, the destruction occurs with the involvement of microfiber and the development of plastic deformations. The development of cracks acquires an unstable, avalanche-like character during the transition from zone 3 to zone 4. An increase of acoustic events per unit of time is observed in the deformation area from zone 4 to zone 6, which is probably due to more intensive crack development. Characteristically, this phenomenon is observed for beam-samples of all mixes with the same deflection in the range from 1.2 to 2.2 mm. Zone 7 is characterized by a more intense release of signals in a sample, which has a more fragile destruction trajectory. This can probably be explained by the interaction of matrix particles with each other, while a sample that has collapsed along a plastic trajectory involves microfibers more effectively.

The proposed approach in substantiating the selected zones makes it possible to obtain tools for indirect inspection and monitoring of building structures, as well as to predict and control the stages of crack development in the bent elements of the structures.

The use of strain-hardening composites based on inorganic binders is actually in structures for which the presence of a strain-hardening zone on the deformation curve of material is of priority importance compared to the value of compressive or bending strength. Walling of mine workings can be one of the areas of application. Often, sprayed concrete with steel mesh reinforcement is used to fasten support. The sprayed concrete support repeats the shape of the mine surface. Dispersed reinforcement with metal or synthetic fiber is performed to improve the mechanical properties of the sprayed concrete support. The main requirement for the mechanical behavior of fiber-reinforced sprayed concrete for walling mine workings is imposed on the post peak zone. The value at which the structure completely loses its ability to resist the load and its destruction occurs are taken as the value of the maximum bearing capacity of the support. Multiple cracking of concrete requires the development of non-destructive methods of structural control in difficult operating conditions [81,82].

The main advantages when using the fiber-reinforced spray concrete for walling the mine workings are as follows [83,84,85,86,87,88]:

- fibers in a large amount contribute to the appearance of strain-hardening of material after the formation of microcracks under external loading;
- mechanical properties of lining increase under complex loading since reinforcement is carried out in all directions of walling;
- residual strength of fiber-reinforced spray concrete is higher than the strength of ordinary concrete;
- connectivity of sprayed concrete with the rock contour increases since there are no voids that can form between the rock contour and the sprayed concrete in the case of mesh reinforcement;
- ensuring the strength properties of concrete under elevated temperature [89,90].

It should be noted that the cost of fiber-reinforced concrete increases significantly with the increase of microfiber amount. The use of ground slags and affordable polypropylene microfiber can solve this problem. Further research should be carried out in order to develop new compositions of strain-hardening composites with inorganic binders based on by-products of the industry including the possibility of applying mixtures using shotcrete technology.

## 5. Conclusions

Strength and deformation properties of pseudo strain-hardening composites based on inorganic binders with polypropylene microfiber under compression and tension in bending with the additional determination of the quantity of acoustic emission signals were carried out. The following new knowledge is obtained in the work: new compositions of strain-hardening material with alkali-activated slag matrix; data on the value of strain-hardening in composites with polypropylene microfiber depending on its quantity and water-to-binder ratio, and data on the number of acoustic signals when approaching the ultimate strength and in the area of residual strength.

A high-strength Portland cement matrix with compressive strength of 70 MPa and alkali-activated slag matrix with a compressive strength of 40 MPa were used. The matrix compositions were selected for high filling the composites with polypropylene microfiber in the amount of 5.5% vol. and 3.5% vol. for Portland cement and alkali-activated slag binder, respectively. Recommendations for the development of new strain-hardening composites with good workability are given.

The increase of PP microfiber amount from 4.4% to 5.5% by volume in Portland cement composites led to a decrease in bending strength. The strain-hardening zone appears on the deformation curves of both mixes. However, this zone in samples with 5.5% vol. of PP microfiber is of interest because the bending strength corresponding to sample destruction exceeds the bending strength corresponding to the first microcrack.

The results show a contradictory pattern in the case of composites with alkali-activated slag matrix. The bending strength decreases with the decrease of the water-to-binder ratio from 0.3 to 0.22 at the constant microfiber amount of 3.5%. The strain-hardening zone appears in composition with W/B = 0.22, which is more pronounced compared to the zone on the deformation curve of composition with W/B = 0.3, namely, the bending strength corresponding to sample destruction exceeds the bending strength corresponding to the first microcrack.

Hydrophobic PP microfibers, in large amounts, reduce the compressive and tensile strength in bending. However, the use of these microfibers in large quantities makes it possible to obtain significant strain-hardening, which is of priority in some areas of construction, for example, in walling of mine workings.

The acoustic emission method was used to assess the destruction of composites. A smaller number of acoustic signals, by 3–4 times, was observed for alkali-activated slag composites. Peaks in the number of acoustic signals in these samples were observed only in the strain-softening zone.

The recorded acoustic signals can be combined into seven groups depending on the stage of sample deformation. The proposed approach in substantiating the selected zones makes it possible to obtain tools for indirect inspection and monitoring of building structures.

## Figures and Tables

**Table 1:** Characteristics of PP microfibers of cited papers.

Microfiber	Tensile Strength, MPa	Fiber Diameter, μm	Fiber Length, mm	Young’s Modulus, GPa	Elongation at Break, %	Density, g/cm^3^	Reference
PP fiber	850	12	10	6	21	0.91	[35]
PP-MF Multi-fiber	>220	20	12	1	100–200	0.91	[40]
PP-SF Split-film fiber	>340	38	20	4	11	0.91	
PP-MF Multi-fiber	910	12	10	9	22	0.91	[41]
PP-Circular	326	25	12	2.74	-	-	[42]
PP-Triangular	247–300	32	12	2.39	-	-	
PP-Trilobal	141–234	38	12	1.25	-	-	
PVA	1600	38	8	42.8	-	-	

**Table 2:** PP microfiber characteristics.

Average diameter	20 µm
Length	6 mm
Shape	round
Density	0.91 g/cm3
Tensile strength	350 MPa
Tensile modulus	5.7 GPa
Elongation at break	250%
Softening temperature	150 °C
Ignition temperature	>320 °C
Water wettability of fiber surface	hydrophobic

**Table 3:** Mix proportions of PP microfiber reinforced Portland cement composite (kg/m^3^).

	Mix 2	Mix 6
Cement CEM I 42.5	1418	1392
Ground quartz sand A1.5	282	282
Quartz sand 0.06–2 mm	145	145
Polycarboxylate-based superplasticizer	35	35
PP microfiber	40	50
Water	312	310
W/C	0.22	0.22
Density after mixing, kg/m^3^	2232	2213
Density at the age of 28 days, kg/m^3^	2120	2070
PP microfiber % by vol.	4.4%	5.5%

**Table 4:** Mix proportions of PP microfiber reinforced alkali-activated slag composite (kg/m^3^).

	Mix 3	Mix 4
Ground granulated blast furnace slag	1133	1275
Ground quartz sand A4	224	232
Quartz sand 0.06–2 mm	113	120
PP microfiber	32	32
Liquid glass with the density of 1.3 g/cm^3^ and with the silicate module equal to 1.5	120	122
NaOH	38	41
Water	338	280
Water-to-slag ratio	0.3	0.22
Density after mixing, kg/m^3^	1998	2102
Density at the age of 28 days, kg/m^3^	1961	2025
PP microfiber % by vol.	3.5%	3.5%

**Table 5:** Compressive strength and tensile strength in bending.

	Compressive Strength, MPa	Standard Deviation, MPa	Coefficient of Variation, %	Tensile Strength in Bending at First Microcrack, MPa	Standard Deviation, MPa	Coefficient of Variation, %
Mix 2 (CEM)	79.59	2.89	4.63	7.93	1.42	17.89
Mix 6 (CEM)	68.06	2.72	3.99	5.81	0.76	13.05
Mix 3 (slag)	42.37	2.86	4.52	3.85	0.56	14.57
Mix 4 (slag)	39.86	2.91	4.21	3.45	0.30	8.60

**Table 6:** Specific fracture work.

Composition.	Mix 2	Mix 6	Mix 3	Mix 4
Compressive strength, MPa	79.59	68.06	42.37	39.86
Tensile strength in bending, MPa (at the first microcrack)	7.93	5.81	3.85	3.45
Specific fracture work, J/m^3^	22,200.4	23,176.9	10,351.5	13,281.2
Strain-hardening after the formation of the first microcrack, MPa	0	0.64	0	0.23
Deflection value, mm	1.15	1.17	1.04	1.08

## Data Availability

Not available.
